# Body Mass Index and Body Fat Percentage of Armed Forces Personnel in Montenegro among Different Age Groups

**Published:** 2020-05

**Authors:** Boris BANJEVIC, Stevo POPOVIC, Bojan MASANOVIC

**Affiliations:** Faculty for Sport and Physical Education, University of Montenegro, Niksic, Montenegro

## Dear Editor-in-Chief

Obesity is recognized as a main problem that increases morbidity and mortality, and international health organizations, such as WHO and IOTF, have labelled obesity as an epidemic-scale disease. the data supporting this claim is reflected in the fact that in 2016, even 1.9 billion adults were over-weight and 650 million suffered from obesity, that represents 39% of overweight and 13% of obese on the level of the entire world adult population (1). If you compare these data with previous, the same source indicated this modern disease has nearly tripled since 1975 and, both theory and practice must deal with it urgently.

Montenegro is no exception when it comes to this issue (2), although there are limitations when it comes to studies of this nature. Obesity in Montenegro is evidently increasing when all populations are concerned, so it is expected that members of the Montenegrin Army will not be bypassed by this issue. Prevention of this chronic non-communicable disease, which represents the second leading cause of premature mortality in the world, right after smoking (1), significantly improves the effectiveness of military service members when it comes to performing professional tasks. Namely, high levels of overweight and obesity, are generally caused by changes in people's lifestyles that is the input in nutrition of groceries with high energy levels and with elements of saturated fat, but also an increasingly inactive lifestyle for all generations. At first, obesity was thought to be a problem for developed countries and urban areas; however, recent studies (2) established that obesity is becoming a global problem for all people in the World, whether they live in rural or urban areas, or in developed or developing countries.

Along with the idea this issue didn’t bypass the Montenegrin soldiers and, the fact body mass index (BMI) and body fat percentage (FAT%) represent significant indicators used in the Montenegrin Army when it comes to health services, the main objective of this study was to do, for the first time, a comprehensive analysis and determine the level of overweight and obesity of members of the military service in relation to their age, first of all because it is expected that this global problem will vary with the age of the indicated population.

The sample of 240 active members of the Armed Forces Personnel in Montenegro included in the analysis were classified into eight age groups: I (18–21 years old), II (22–26), III (27–31), IV (32–36), V (37–41), VI (42–46), VII (47–51) and VIII (50+). BMI and FAT% were calculated according to the body structure assessment protocols, provided in the ACSM'S Health-Related Physical Fitness Assessment Manual of the American College of Sports Medicine (ACSM). Descriptive statistics were used to calculate demographic and body composition characteristics, while a one-way ANOVA and Post Hoc test were used with purpose to determine differences between age groups. The significance level was set at p <0.05. The mean age, height, weight, BMI and FAT% of the subjects were 36.93 years old, 181.29 cm, 91.95 kg, 28.00 kg/m^2^ and 11.70% respectively, while the age groups have specific mean BMI and FAT%: I (24.58 kg/m^2^ and 5.42%), II (26.11 kg/m^2^ and 7.59%), III (27.72 kg/m^2^ and 9.33%), IV (28.49 kg/m^2^ and 12.04%), V (28.94 kg/m^2^ and 13.70%), VI (28.63 kg/m^2^ and 13.98%), VII (29.55 kg/m^2^ and 14.78%) and VIII (29.95 kg/m^2^ and 16.78%). Hence, based on the BMI classification of WHO, out of 240 tested subjects in this study, no subjects were underweight (<18.50 kg/m^2^), while 51 subjects (21.3%) were normal (18.50–24.99 kg/m^2^), 109 subjects (45.5%) were overweight (25.00–29.99 kg/m^2^) and 58 subjects (24.2%) obese (≥30.00 kg/m^2^). On the other hand, judging from the age perspective, just first group showed the normal range, while all other age groups were approaching 25.0 (overweight or pre-obese) and more, but not over 30.0 (obese) on average. Furthermore, based on the FAT% normative of ACSM, the contrary results were reached. The youngest age group was described as “excellent”, while all others were “very good” that was unexpected finding. It raises new and support previous research questions when it comes to WHO normative and its application in the Western Balkan’s populations (3) and its specific body composition. The ANOVA were employed and showed significant differences on both tested variables, while Post Hoc test did not show significant differences on all age groups, except for subjects’ BMI and FAT% between the first two groups (I: 18–21 and II: 22–26 years old) and the rest, that corresponds to expected assumptions.

In conclusion, judging from the BMI perspective, this study have an alarming prevalence of over-weight (45.5%) and obesity (24.2%) that may lead Armed Forces Personnel in Montenegro to adverse health consequences and cause diseases, such as hypertension, cardiovascular ailments, and type II diabetes mellitus, as well as decrease of their physical fitness and effectiveness of military service. However, judging from the FAT% perspective, the findings suggest that Montenegro is not facing an increasing problem of overweight among its military personnel as all subjects were classified under recommended categories. Thus, such findings again raise the suspicion that BMI cannot be applied in the Western Balkans, since the specificity of the body composition can affect disproportionately increased BMI at the expense of the muscular and bony components and requires to create specific normative for this region in the further investigations.
